# Dynamic Sensing Performance of a Point-Wise Fiber Bragg Grating Displacement Measurement System Integrated in an Active Structural Control System

**DOI:** 10.3390/s111211605

**Published:** 2011-12-13

**Authors:** Kuo-Chih Chuang, Heng-Tseng Liao, Chien-Ching Ma

**Affiliations:** 1 School of Aeronautics and Astronautics, Institute of Applied Mechanics, Zhejiang University, Hangzhou 310027, China; E-Mail: chuangkc@zju.edu.cn; 2 Department of Mechanical Engineering, National Taiwan University, Taipei 106, Taiwan; E-Mail: r95522503@ntu.edu.tw

**Keywords:** fiber Bragg grating, sensing system, out-of-plane, point-wise displacement sensor, amplitude-fluctuation electronic speckle pattern interferometry, smart cantilever beam, velocity feedback control

## Abstract

In this work, a fiber Bragg grating (FBG) sensing system which can measure the transient response of out-of-plane point-wise displacement responses is set up on a smart cantilever beam and the feasibility of its use as a feedback sensor in an active structural control system is studied experimentally. An FBG filter is employed in the proposed fiber sensing system to dynamically demodulate the responses obtained by the FBG displacement sensor with high sensitivity. For comparison, a laser Doppler vibrometer (LDV) is utilized simultaneously to verify displacement detection ability of the FBG sensing system. An optical full-field measurement technique called amplitude-fluctuation electronic speckle pattern interferometry (AF-ESPI) is used to provide full-field vibration mode shapes and resonant frequencies. To verify the dynamic demodulation performance of the FBG filter, a traditional FBG strain sensor calibrated with a strain gauge is first employed to measure the dynamic strain of impact-induced vibrations. Then, system identification of the smart cantilever beam is performed by FBG strain and displacement sensors. Finally, by employing a velocity feedback control algorithm, the feasibility of integrating the proposed FBG displacement sensing system in a collocated feedback system is investigated and excellent dynamic feedback performance is demonstrated. In conclusion, our experiments show that the FBG sensor is capable of performing dynamic displacement feedback and/or strain measurements with high sensitivity and resolution.

## 1. Introduction

Fiber Bragg grating (FBG) sensors possess many excellent properties such as small size, mass-production at low cost, and immunity to electro-magnetic interference (EMI). For more than a decade, they have been proven to be sensitive to many physical quantities such as strain, temperature, pressure, acceleration, and force [1–3]. Since many FBGs can be inscribed into a single fiber, they also have multiplexing ability to detect several different positions in the same structure simultaneously. Traditionally, FBG sensors are mounted on or imbedded in structures. Thus, detecting out-of-plane point-wise displacement can’t be achieved without work-around methods such as bonding an FBG sensor to a cantilever structure to indirectly measure the displacement responses [4,5]. However, indirect sensing methods are still not capable of measuring point-wise displacement. Due to the different modes of the cantilever structures, indirect sensing methods are also not suitable for dynamic measurements.

To directly utilize an FBG to measure out-of-plane point-wise displacement, a method that can point-wisely glue an FBG sensor perpendicular to the surface containing the detecting point is adopted in this paper [6]. Since sensors are one of the key elements in smart structures [7], the purpose of this work is to investigate the feasibility of integrating the proposed FBG displacement sensing system into smart structures for performing active vibration suppression. A smart cantilever beam actuated by a piezoelectric actuator is employed to demonstrate the dynamic sensing ability as well as the feedback sensing ability of the proposed FBG displacement sensing system.

The sensing principle of the FBG is based on the shift of the Bragg wavelength resulting from variations of environmental physical quantities. Under static or low frequency condition, an optical spectrum analyzer (OSA) is usually used to detect wavelength shift [8,9]. However, since the demodulating speed of the OSA is limited, wavelength shifts are not able to be recorded in time for high frequency signals. On the contrary, demodulating techniques which transfer wavelength shifts to energy variations through optical filters are capable of detecting high frequency responses. Optical filters such as the long-period fiber grating (LPFG) filters, FBG filters, and chirped FBG filters are capable of providing different dynamic sensing ranges and resolutions [10–12]. Among above mentioned filters, the FBG filter can provide the highest sensitivity and it possesses high signal-to-noise (SNR) ratio due to its smallest full-width at half maximum (FWHM) compared to other grating-based filters. Thus, different from the sensing system in [6] which employs an LPFG filter to demodulate the displacement responses, this study employs an FBG filter in the sensing system to enhance the dynamic sensing performance.

There are several control algorithms that have been successfully utilized to control flexible smart structures [13]. Since the purpose of this study is focused on the dynamic sensing performance of the proposed point-wise FBG displacement sensor system in an active structural control application, a velocity feedback control is adopted in this paper to add damping of the smart cantilever beam and suppress the vibrations. The proposed FBG displacement sensor and the piezoelectric actuator is set up collocated to each other on top and bottom surfaces of the cantilever beam, respectively. Before performing experiments, an optical full-field measurement technique called amplitude-fluctuation electronic speckle pattern interferometry (AF-ESPI) is used to provide full-field vibration mode shapes and resonant frequencies. To verify the dynamic demodulation performance of the FBG filter, a traditional FBG strain sensor calibrated with a strain gauge is first employed to measure the dynamic strain of impact-induced vibrations. Then, system identification of the smart cantilever beam is performed by FBG strain and displacement sensors. To verify dynamic sensing ability of the proposed FBG displacement sensor, a laser Doppler vibrometer (LDV) is simultaneously employed as a comparison for the displacement measurement. Finally, since the effect of the velocity feedback for sinusoidal waveforms is equivalent to delay the waveforms by some phases, a delay controller is also utilized in our work as a comparison for the velocity feedback controller. To our knowledge, this is the first time that the proposed FBG displacement sensing system being integrated into a smart structure for performing active vibration control.

This paper is organized as follows. Section 2 summarizes the sensing principle, calibration method, and set-up method of the FBG displacement sensor. The model of the smart cantilever beam is presented in Section 3. The velocity feedback controller and the delay controller are briefly introduced in Section 4. Finally, the experimental setup, performances of the FBG-filter based demodulation technique, system identifications performed by FBG strain/displacement sensors, and control results of suppressing vibrations of cantilever beam are reported and discussed in Section 5.

## 2. FBG Sensing System

A fiber Bragg grating (FBG) is a periodic distribution of the refractive index along the fiber core. From the Bragg’s law, the Bragg wavelength *λ*_S_ of an FBG sensor is given by [14]:

(1) $$\lambda_{S}=2n_{\text{eff}}\Lambda$$

where Λ is the Bragg grating period and *n*_eff_ is the effective refractive index of the fiber core. The shift in Bragg wavelength due to an applied strain can be expressed as:

(2) $$\Delta \lambda_{S}=(1-p_{e})\lambda_{S0}\varepsilon$$

where *ε* is the strain induced in the fiber, *p_e_* is the effective photoelastic coefficient, and *λ*_S0_ is the Bragg wavelength of the grating without the strain field. In order to measure the point-wise displacement of a point on a structure subjected to dynamic loadings using an FBG, one end of a fiber (of length *l*_0_) containing the FBG needs to be fixed to a stationary boundary while the other end is fixed to the sensing point. The dynamic displacement of the sensing point on the structure can be obtained from the following relation:

(3) $$d(t)=\int_{0}^{l_{0}}\varepsilon (t)dz$$

The spectrum of the light reflected by the FBG sensor can be approximated by a Gaussian function, given by:

(4) $$R(\lambda )=R_{S}\;\text{exp}\left[-4\;\text{ln}2{\left(\frac{\lambda -\lambda_{S}}{\sigma_{S}}\right)}^{2}\right]$$

where *R*_S_ is the maximum reflectivity of the FBG sensor, *σ*_S_ is the grating full-width at half maximum (FWHM). In order to enhance the signal-to-noise (SNR) ratio and the sensitivity, an FBG filter located at the output of a broadband source (BBS) is used as a demodulator in the sensing system. If the intensity of the BBS is *P*(*λ*), the total light power *I* detected by the photodetector (PD) can be expressed as:

(5) $$I=\int_{-\infty }^{\infty }P(\lambda )F(\lambda )R(\lambda )d\lambda ≅P_{\lambda }\int_{-\infty }^{\infty }F(\lambda )R(\lambda )d\lambda$$

where *F*(*λ*) is the spectrum of the FBG demodulator. For a broadband source with a relative flat spectrum, the power spectrum may be assumed to be a constant as *P_λ_* over the operation range. The PD transforms the light intensity to voltage signals.

Before performing the measurement, calibration of the FBG sensing system is necessary to ensure the linearity, maximum sensing output, and sensitivity. The calibrating criteria are based on the demodulation behavior of the FBG filter. The transmittance of the FBG filter can be approximated by [14–16]:

(6) $$F(\lambda )=1-R_{F}\;\text{exp}\left[-4\;\text{ln}2{\left(\frac{\lambda -\lambda_{F}}{\sigma_{F}}\right)}^{2}\right]$$

where *λ*_F_ is the Bragg wavelength of the filter, *R*_F_ is the maximum reflectivity, and *σ*_F_ is the grating FWHM of the FBG filter. Substituting Equations (4) and (6) into Equation (5), the light intensity after evaluating the integration is expressed as:

(7) $$I=I_{\text{DC}}-I_{P}\;\text{exp}\left[-{\left(\delta \lambda_{\text{nor}}\right)}^{2}\right]$$

In Equation (7), *I*_DC_ is the DC component of the light source given by:

(8) $$I_{\text{DC}}=\frac{\sqrt{\pi }P_{\lambda }R_{S}\sigma_{S}}{\sqrt{4\;\text{ln}\;2}}$$

*I*_p_ is the transmittance light intensity given by:

(9) $$I_{P}=\frac{\sqrt{\pi }P_{\lambda }R_{F}R_{S}}{\sqrt{4\;\text{ln}2}}\frac{\sigma_{F}\sigma_{S}}{\sqrt{\sigma_{F}^{2}+\sigma_{S}^{2}}}$$

and *δλ*_nor_ is a normalized wavelength mismatch defined as:

(10) $$\delta \lambda_{\text{nor}}=\left(\frac{2\sqrt{\text{ln}2}}{\sqrt{\sigma_{F}^{2}+\sigma_{S}^{2}}}\right)\delta \lambda$$

where *δλ* = *λ*_S_ − *λ*_F_ = *λ*_S0_ − *λ*_F_ + Δ*λ*_S_ is the wavelength mismatch. Hence, the variation of intensity *I* will depend on the wavelength mismatch. It is noted that if *λ*_F_ of the FBG filter attached to a translation stage is adjusted to the left of *λ*_S_ before the measurement, the PD output electrical signal will increase as the FBG sensor is elongated and decrease as the FBG sensor is compressed. For the case that small dynamic strain is applied, the response of the FBG sensor is linear and the wavelength-to-intensity conversion factor *K* depending on the operation point can be derived from Equation (7) as:

(11) $$K=\frac{dI}{d(\delta \lambda_{\text{nor}})}=2I_{P}\delta \lambda_{\text{nor}}\;\text{exp}\left[-{\left(\delta \lambda_{\text{nor}}\right)}^{2}\right]$$

The maximum wavelength-to-intensity conversion factor *K*_nor,max_ can be obtained by setting *dK*_nor_/d(*δλ*_nor_) = 0, where *K*_nor_ = *K/*2*I*_P_. The normalized wavelength mismatch at *K*_nor,max_ is $1/\sqrt{2}$ which indicates that an optimal operation point of *δλ* is:

(12) $$\delta \lambda_{\text{OPT}}=\lambda_{S0}-\lambda_{F}+\Delta \lambda_{S,\text{OPT}}=\sqrt{\frac{\sigma_{F}^{2}+\sigma_{S}^{2}}{8\;\text{ln}2}}$$

If the optimal operation condition, *i.e.*, *δλ*_OPT_, is matched, then the maximized PD output can be obtained. Meeting of the optimal operation condition is a method to calibrate the FBG sensor before the experiment. An optical spectrum analyzer (OSA) can be employed to monitor the optimal operation condition during the experiment. In this paper, the proposed FBG displacement sensor with high sensitivity is used to detect and feedback the displacement responses of a smart cantilever beam. Since the cantilever beam is actuated by a piezoelectric actuator near the fixed end of the cantilever beam, we can apply a sinusoidal voltage input to the piezoelectric actuator at the first resonant frequency of the cantilever beam to excite the vibration and slightly adjust the translation stage of the FBG filter to prevent the displacement waveform from saturation.

**Figure 1.**
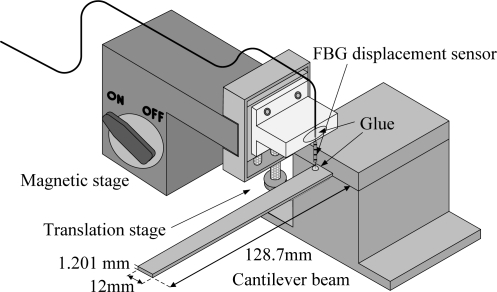
Illustration for the set-up method for the displacement FBG sensor.

To set up an FBG as an out-of-plane FBG displacement sensor, one end of the FBG displacement sensor is glued to a vertical translation stage and the other end of the FBG is glued to the sensing point on the surface of the cantilever beam with a mix of epoxy resin and hardener. Since FBGs are very sensitive to the longitudinal strain, the set-up method mentioned above allows an FBG to be capable of measuring the out-of-plane dynamic displacement of the cantilever beam with high sensitivity. The illustration of the set-up method for the proposed FBG displacement sensor is shown in Figure 1. Details regarding the FBG displacement sensor set-up method can be found in Chuang and Ma [6].

## 3. Model of the Smart Cantilever Beam

In this section, the model of the smart cantilever beam is briefly presented [17–19]. Due to the fact that the proposed point-wise FBG displacement sensor is very sensitive, it is attached near the fixed end of the cantilever beam to avoid saturations of measurement results. Since the thickness is small compared to the length and width of the cantilever beam, the effects of shear deformation and rotary inertia can be neglected. First, consider the free strain *ε_a_* of the actuating layer when a voltage *v_a_* is applied to a piezoelectric material polarized in *z* direction as:

(13) $$\varepsilon_{a}=\frac{d_{31}}{t_{a}}v_{a}$$

where *d*_31_ is the piezoelectric constant of the piezoelectric material, *t*_a_ is the thickness of the piezoelectric actuator. Assume bonding between the actuator and the beam is perfect and the strain distribution is linear across the thickness of the beam, the strain distribution can be decomposed into flexural component and longitudinal component as:

(14) $$\varepsilon (y)=\alpha y+\varepsilon_{0}$$

The stress distribution inside the piezoelectric actuator is:

(15) $$\sigma_{a}(y)=E_{a}(\alpha y+\varepsilon_{0}-\varepsilon_{a})$$

where *E_a_* is the Young’s modulus of the piezoelectric material. The stress distribution inside the beam is:

(16) $$\sigma_{b}(y)=E_{b}\alpha y$$

where *E_b_* is the Young’s modulus of the beam. By applying the moment equilibrium about the center of the beam:

(17) $$\int_{-t_{b}/2}^{t_{b}/2}\sigma_{b}(y)ydy+\int_{t_{b}/2}^{t_{b}/2+t_{a}}\sigma_{a}(y)ydy=0$$

and the force equilibrium along the longitudinal axis of the beam:

(18) $$\int_{-t_{b}/2}^{t_{b}/2}\sigma_{b}(y)dy+\int_{t_{b}/2}^{t_{b}/2+t_{a}}\sigma_{a}(y)dy=0$$

we can obtain *ε*_0_ and *a*, in which:

(19) $$\alpha =\frac{6E_{b}E_{a}t_{b}t_{a}(t_{b}+t_{a})}{E_{b}^{2}t_{b}^{4}+E_{a}E_{b}(4t_{b}^{3}t_{a}+6t_{b}^{2}t_{a}^{2}+4t_{b}t_{a}^{3})+E_{a}^{2}t_{a}{}^{4}}\varepsilon_{a}$$

where *t_b_* is the thickness of the beam. The induced moment *M* (*x*, *t*) in the beam can be expressed as:

(20) $$M(x,t)=E_{b}I_{b}\alpha =C_{a}v_{a}(x,t)=C_{a}v_{a}(t)[u(x-x_{1})-u(x-x_{2})],$$

where *u* stands for the unit step function, *x*_1_ and *x*_2_ are coordinates of the left and right ends of the piezoelectric actuator, and *I_b_* is the moment of inertia of the beam and:

(21) $$C_{a}=\frac{6d_{31}I_{b}E_{b}^{2}E_{a}t_{b}(t_{b}+t_{a})}{E_{b}^{2}t_{b}^{4}+E_{a}E_{b}(4t_{b}^{3}t_{a}+6t_{b}^{2}t_{a}^{2}+4t_{b}t_{a}^{3})+E_{a}^{2}t_{a}{}^{4}}$$

From the Euler-Bernoulli beam theory, the governing equation of motion of the cantilever beam actuated by a piezoelectric patch is given by:

(22) $$\frac{\partial^{2}}{\partial x^{2}}\left[E_{b}I_{b}\frac{\partial^{2}w(x,t)}{\partial x^{2}}\right]+\rho A_{b}\frac{\partial^{2}w(x,t)}{\partial t^{2}}=C_{a}\frac{\partial^{2}v_{a}(x,t)}{\partial x^{2}}$$

where *w*(*x*,*t*) is the out-of-plane displacement of the beam from its equilibrium, *ρ* is the mass density, and *A_b_* is the cross-sectional area of the beam. The boundary conditions of the cantilever beam are given by:

(23) $$w(0,t)=\frac{\partial }{\partial x}w(0,t)=\frac{\partial^{2}}{\partial x^{2}}w(L,t)=\frac{\partial^{3}}{\partial x^{3}}w(L,t)=0$$

where *L* is the length of the cantilever beam. The out-of-plane displacement *w*(*x*,*t*) can be expanded as an infinite series of eigenfunctions in the form:

(24) $$w(x,t)=\sum_{i=1}^{\infty }\phi_{i}(x)q_{i}(t)$$

where *φ_i_*(*x*) is the mode shape, also known as eigenfunction, satisfying the ordinary differential equations. From the following orthogonality properties:

(25) $$\int_{0}^{L}\phi_{i}(x)\phi_{j}(x)dx=\delta_{ij}$$

(26) $$\int_{0}^{L}\frac{E_{b}I_{b}}{\rho A_{b}}\frac{d^{4}}{dx^{4}}\phi_{i}(x)\phi_{j}(x)dx=\omega_{i}^{2}\delta_{ij}$$

where *δ_ij_* is the Kronecker delta function. From the boundary conditions of the cantilever, the mode shape is given by:

(27) $$\varphi_{i}(x)=C_{i}\;\left[\text{cosh}\;\beta_{i}x-\text{cos}\;\beta_{i}x-\frac{\text{cos}\;\beta_{i}x+\text{cosh}\;\beta_{i}x}{\text{sin}\;\beta_{i}x+\text{sinh}\;\beta_{i}x}\left(\text{sinh}\;\beta_{i}x-\text{sin}\;\beta_{i}x\right)\right]$$

where *β_i_* are the roots of the following characteristic equation:

(28) $$1+\text{cos}\;\beta_{i}x\;\text{cosh}\;\beta_{i}x=0$$

The resonant frequencies *ω_i_* of the smart cantilever beam are evaluated from $\omega_{i}=\sqrt{\frac{E_{b}I_{b}}{\rho A_{b}}}\beta_{i}^{2}$. Multiplying the governing equation of the Euler-Bernoulli beam by *φ_j_*(*x*) and integrating over the length of the beam, we have:

(29) $$\frac{E_{b}I_{b}}{\rho A_{b}}\int_{0}^{L}\sum_{i=1}^{\infty }q_{i}(t)(\frac{d^{4}}{dx^{4}}\varphi_{i}(x))\varphi_{j}(x)dx+\int_{0}^{L}\sum_{i=1}^{\infty }{\ddot{q}}_{i}(t)\varphi_{i}(x)\varphi_{j}(x)dx=\frac{1}{\rho A_{b}}\int_{0}^{L}C_{a}\frac{\partial^{2}v_{a}(x,t)}{\partial x^{2}}\varphi_{j}(x)dx$$

From the orthogonality properties Equations (25) and (26), the property of Dirac delta function, and with the assumption that the voltage *v_a_*(*x*,*t*) is constant in the range [*x*_1_, *x*_2_], we have:

(30) $$\begin{array}{lll} \left({\ddot{q}}_{i}(t)+\omega_{i}{}^{2}q_{i}(t)\right) & =\frac{1}{\rho A_{b}}\int_{0}^{L}C_{a}\frac{\partial^{2}v_{a}(x,t)}{\partial x^{2}}\varphi_{i}(x)dx\; & \\ & =\frac{1}{\rho A_{b}}\int_{0}^{L}C_{a}[\delta^{\prime }(x-x_{1})-\delta^{\prime }(x-x_{2})]v_{a}(t)\varphi_{i}(x)dx & \\ & =\frac{C_{a}}{\rho A_{b}}[\varphi_{i}^{\prime }(x_{2})-\varphi_{i}^{\prime }(x_{1})]v_{a}(t) & i=1,...,\infty \end{array}$$

By adding natural damping of the beam, the governing equation *q_i_*(*t*) for the smart cantilever beam can be written as follows:

(31) $$\begin{array}{ll} {\ddot{q}}_{i}(t)+2\varsigma_{i}\omega_{i}{\dot{q}}_{i}(t)+\omega_{i}{}^{2}q_{i}(t)=\frac{C_{a}}{\rho A_{b}}[\varphi_{i}^{\prime }(x_{2})-\varphi_{i}^{\prime }(x_{1})]v_{a}(t) & \;\;\;i=1,...,\infty \end{array}$$

In this study, a simple but effective negative velocity feedback controller is employed to suppress vibrations of the smart cantilever beam as well as to demonstrate dynamic sensing performance of the proposed FBG displacement sensor system. In velocity feedback control, the displacement responses obtained by the FBG sensor are differentiated and fed back to the piezoceramic actuator. The structure of the velocity feedback controller can be written as follows:

(32) $$v_{a}(t)=-G{\dot{q}}_{i}(t)$$

where *G* is the constant control gain. Equation (31) can then be rewritten as:

(33) $$\begin{array}{ll} {\ddot{q}}_{i}(t)+\left(2\varsigma_{i}\omega_{i}+G\frac{C_{a}}{\rho A_{b}}[\varphi_{i}^{\prime }(x_{2})-\varphi_{i}^{\prime }(x_{1})]\right){\dot{q}}_{i}(t)+\omega_{i}{}^{2}q_{i}(t)=0 & \;\;\;i=1,...,\infty \end{array}$$

Thus, it is obvious that velocity feedback control has the effect to add damping of the flexible structure and can be used to suppress the vibration.

**Figure 2.**
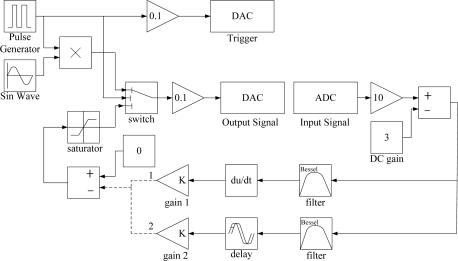
Simulink program for active vibration control.

In this study, the velocity feedback controller is developed in Simulink and implemented by a dSPACE DS1104 system with a sampling frequency set to 50 kHz. Figure 2 shows the Simulink program in which the exciting and control process are combined into one program. First the smart cantilever beam is excited by the piezoceramic actuator to the steady state at the first or second natural frequency. During the steady-state vibration, actuator’s input is terminated suddenly to induce free vibration. At the same time, feedback loop is connected to apply control algorithms to suppress the vibration of the cantilever beam.

## 4. Experimental Results and Discussion

**Figure 3.**
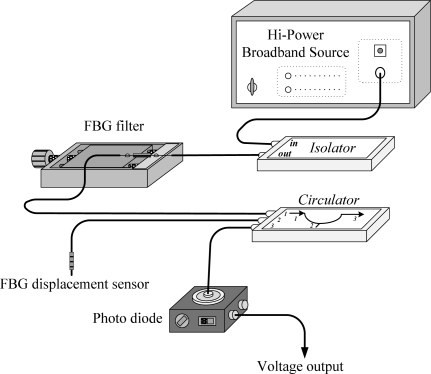
Schematic diagram for the FBG displacement sensing system.

**Figure 4.**
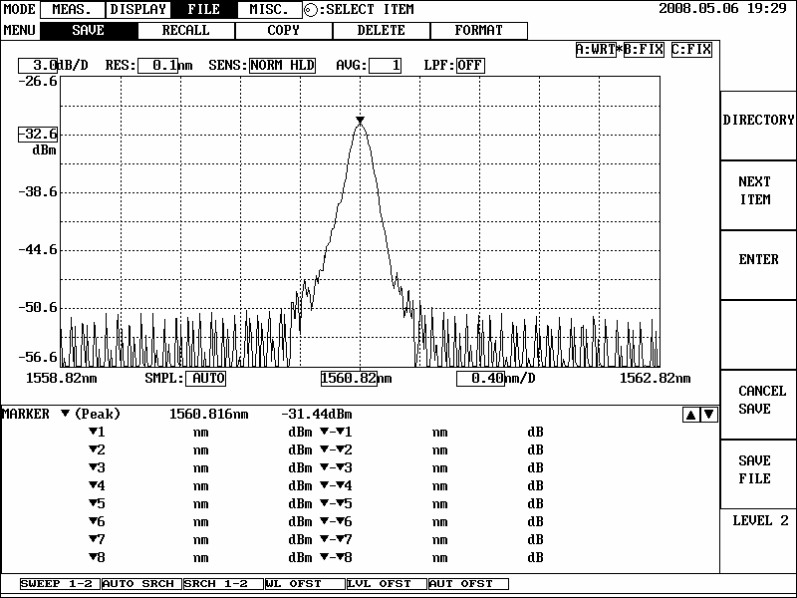
Reflection spectrum of the FBG displacement sensor.

**Figure 5.**
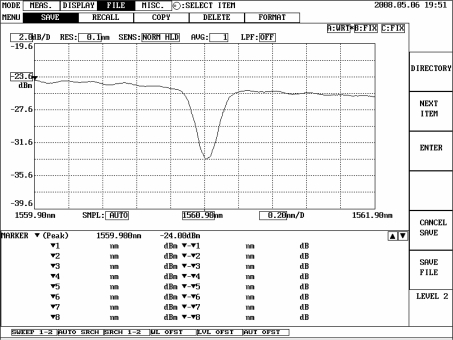
Transmittance spectrum of the FBG filter.

Figure 3 shows the schematic diagram of the FBG sensing system. A broadband source (BBS) with a wavelength ranging from 1,520 to 1,570 nm transmits light to an isolator, which is used to prevent the light from reflecting back to the light source. Then the light beam enters an FBG filter to form a transmittance spectrum. The FBG filter is attached to a translation stage so that the dip transmittance of the filter can be adjusted to meet the optimal demodulation condition. Finally the light beam is coupled to a directional circulator and reflected back to the corresponding photodetector (PDA10CS, InGaAs amplified detector, Thorlabs, Inc.) on the output channel. The electrical signals are fed back into the dSPACE DS1104 hardware to perform the active vibration suppression. The original Bragg wavelength of the FBG sensor is 1,560.82 nm. The FBG sensor used in our experiment is fabricated with FWHM of approximately 0.13 nm and reflectivity of less than 90% to avoid distortions and saturations of the responses. The FBG sensor and FBG filter are both fabricated with grating lengths of 10 mm. The reflection spectrum of the FBG sensor and the transmittance spectrum of the FBG filter are shown in Figures 4 and 5, respectively.

The cantilever beam used in the experiment is made of 6,061 aluminum. The dimensions of the cantilever beam are shown in Figure 1. The actuator used in this study is an APC 855 piezoceramic plate and its parameters are shown in Table 1. Before performing the sensing and feedback experiments, we employ an optical amplitude-fluctuation electronic speckle pattern interferometry (AF-ESPI) [20] technique to provide full-field vibration mode shapes as well as the resonant frequencies of the smart cantilever beam. Compared to the ordinary time-averaging method, the fringe patterns obtained by the AF-ESPI method largely enhance visibility and reduce noise. When the vibration frequency of the smart cantilever beam is near the resonant frequency, stationary distinct fringe patterns will be observed in a monitor. Thus, the AF-ESPI can be used to simultaneously obtain full-field vibration mode shapes as well as resonant frequencies. The mode shapes and resonant frequencies of the smart cantilever beam obtained by the AF-ESPI technique are shown in Tables 2 and 3 for the bending modes and the torsional modes, respectively. The brightest lines in AF-ESPI measurements are the nodal lines of the vibration mode shapes.

In this study, a non-contact laser Doppler vibrometer (LDV) which can measure point-wise out-of-plane displacement responses is employed simultaneously to compare measurement results obtained from the proposed FBG displacement senor.

**Figure 6.**
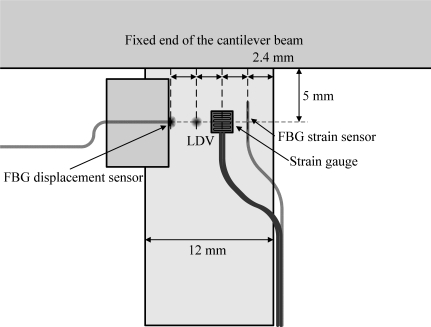
Illustration of locations of the sensors on the cantilever beam.

A traditional FBG strain sensor and a strain gauge are also used to verify the performance of the FBG filter before performing the experiments for the FBG displacement sensor. The sensing locations of the FBG displacement sensor, LDV, FBG strain sensor, and strain gauge are shown in Figure 6.

**Figure 7.**
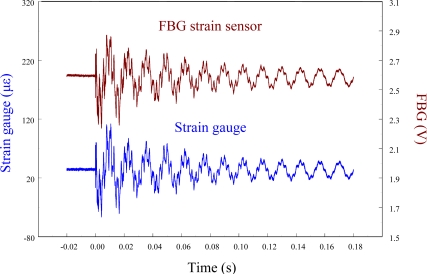
Transient strain responses of the smart cantilever beam subjected to the impact loading by a steel ball.

**Figure 8.**
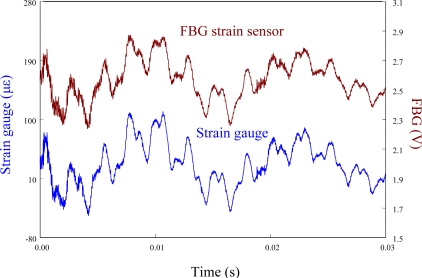
Transient strain responses of the smart cantilever beam subjected to the impact loading by a steel ball within 0.03 s.

The performance of the FBG filter is first demonstrated by the strain sensors (*i.e.*, FBG strain sensor and strain gauge). A steel ball with 3.8 mm in diameter is dropped on the centerline and 10 mm away from the free end to induce transient wave propagation in the cantilever beam. Figure 7 demonstrates the measured results of the transient strain responses obtained by the traditional FBG strain sensor and strain gauge simultaneously. To observe the strain responses in detail, Figure 7 is replotted within 0.03 s and the results are shown in Figure 8. Since the transient strain responses obtained by the two strain sensors agree well with each other in Figure 8, the FBG is proved to be capable of measuring dynamic strain with high resolution both in time and space. From Figures 7 and 8, we can see that the proposed FBG filter offers excellent dynamic demodulation performance.

**Figure 9.**
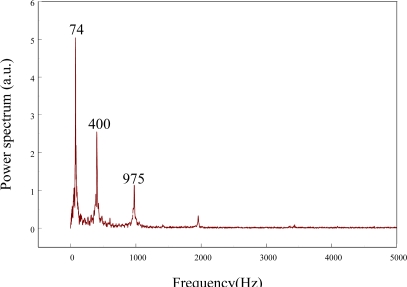
Frequency spectrum of the smart cantilever beam.

The frequency spectrum of the smart cantilever beam can be constructed from taking the fast Fourier transform of the transient strain responses obtained by the FBG strain sensor and the results are shown in Figure 9. Next, we excite the piezoelectric actuator with random inputs to perform system identification by the stochastic spectral estimation. The random signal input is generated by dSPACE DS1104 system with the sampling frequency set to 50 kHz. From the input and output data recorded by dSPACE DS1104 system, the frequency response function (FRF) of the system is determined from the relation [21]:

(34) $$G(j\omega )=\frac{S_{\mathrm{yu}}(j\omega )}{S_{\mathrm{uu}}(j\omega )}$$

where *S_uu_* (*jω*) is the auto-spectral density function of the input random signal and *S_yu_* (*jω*) is the cross-spectral density function of input and output signals (*i.e.*, responses obtained by the FBG strain sensor).

**Figure 10.**
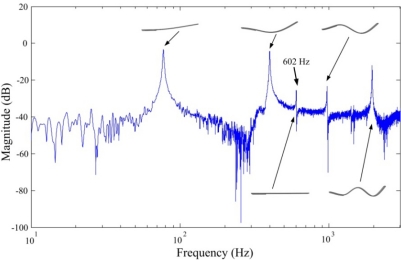
Frequency response of the smart cantilever beam obtained by the FBG strain sensor.

**Figure 11.**
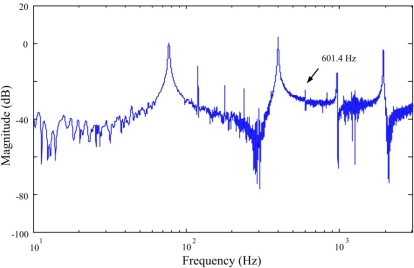
Frequency response of the smart cantilever beam obtained by the strain gauge.

The frequency responses obtained by the FBG strain sensor and strain gauge are shown in Figures 10 and 11, respectively.

From both Figures 10 and 11, we can see that a lateral mode (*i.e.*, 602 Hz in Figure 10 and 601.4 Hz in Figure 11) is measured by the two sensors. However, frequency response obtained by the conventional strain gauge (*i.e.*, Figure 11) contains more measurement noises especially between the first and the second bending modes of the smart cantilever beam. The identified first three bending modes of the smart cantilever beam without the presence of the steel ball are 76 Hz, 400 Hz, and 975 Hz, respectively. Since the error between the first resonant frequency is quite small, it is acceptable to say that the resonant frequencies can be obtained from the impact-induced transient responses. However, for the purpose of active vibration control, the first bending mode of the smart cantilever beam is considered to be 76 Hz in this work.

**Figure 12.**
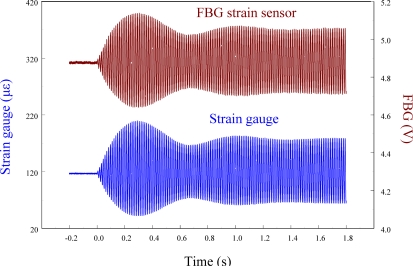
Transient strain responses of the smart cantilever beam excited by the piezoelectric actuator at 76 Hz.

We further investigate the dynamic demodulation ability of the FBG filter and the response time for the piezoceramic actuator to achieve the steady state vibration condition. The smart cantilever beam is excited by the piezoelectric actuator at the first resonant frequency (76 Hz) and the second resonant frequency (400 Hz). Figure 12 demonstrates the dynamic strain from transient response to steady state of the smart cantilever beam excited at 76 Hz.

**Figure 13.**
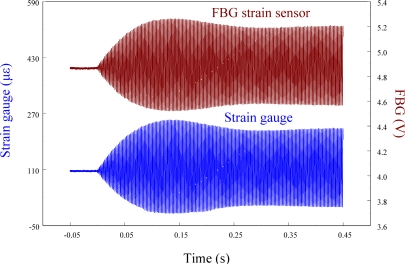
Transient strain responses of the smart cantilever beam excited by the piezoelectric actuator at 400 Hz.

From Figure 12, we can see that there is an overshoot in the transient strain responses before reaching the steady state. The response time from the beginning of excitation to the steady state is about 1.26 s. The transient strain responses of the smart cantilever beam excited at the second resonant frequency (*i.e.*, 400 Hz) is demonstrated in Figure 13. The response time in this case is about 0.33 s. The same phenomenon of overshoot, although is smaller, can still be observed in the transient strain responses excited at 400 Hz.

**Figure 14.**
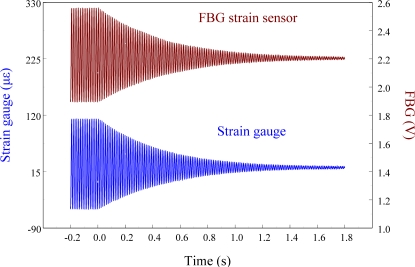
Free vibration of the smart cantilever beam excited at first resonant frequency (76 Hz) obtained from FBG strain sensor and strain gauge.

Figure 14 shows the sensing results obtained from two strain sensors simultaneously when the smart cantilever beam vibrates freely after excitation at 76 Hz is removed.

**Figure 15.**
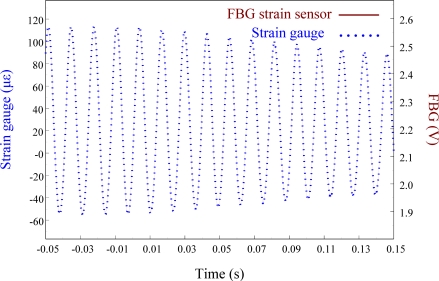
Free vibration of the smart cantilever beam excited at first resonant frequency (76 Hz) with a span of 0.2 s.

**Figure 16.**
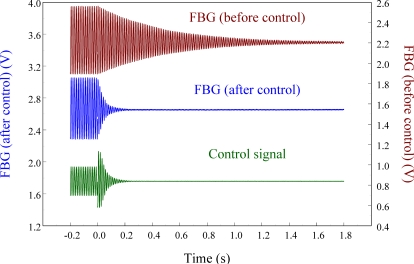
Strain response of cantilever beam excited at first natural frequency (76 Hz) under velocity feedback control.

Figure 15 overlaps the two results and focuses on the responses with a span of 0.2 s. Since the responses measured from these two strain sensors correspond excellently with each other, we can again see that the FBG filter is capable of demodulating responses obtained from the FBG sensor dynamically. Finally, to see the demodulation effect of the FBG filter in an active vibration control system, the negative velocity feedback controller is utilized. As shown in Figure 16, with response before control and the control signal as comparisons, the control result obtained from the FBG strain sensor approaches to zero at about 0.3 s. Since the control effect shown in Figure 16 agrees well with the prediction that the velocity feedback can add damping of the cantilever beam, the dynamic demodulation performance of the FBG filter in an active vibration control system is demonstrated.

**Figure 17.**
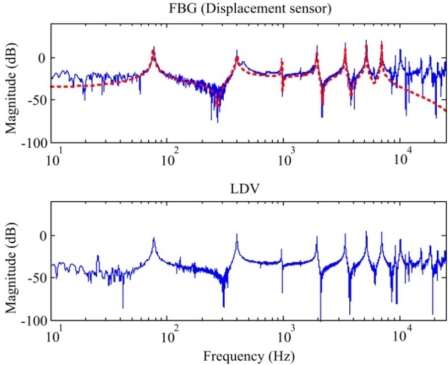
Frequency responses obtained by the FBG displacement sensor and LDV.

Now we can focus on the dynamic sensing performance of the proposed out-of-plane FBG displacement sensor. First, we perform the system identification of the smart cantilever beam again by the stochastic spectral estimation with the FBG displacement sensor and a non-contact laser Doppler vibrometer (LDV). Since the LDV has high sensitivity and resolution, its measurement results are used for comparisons with the results obtained by the FBG displacement sensor. The obtained frequency responses obtained by the two sensors are shown in Figure 17. A fourteenth model obtained from the concept of constructing the Bode plot is obtained and represented as dashed lines, as shown in Figure 17. Thus, we can see that the proposed FBG displacement sensing system can be utilized to perform system identification for the smart cantilever beam. In Figure 17, the frequency responses obtained by the FBG and LDV agree well with each other except for the low frequencies below the first bending mode. In fact, the discrepancies for low frequencies are due to the length of the random inputs recorded in the computer.

**Figure 18.**
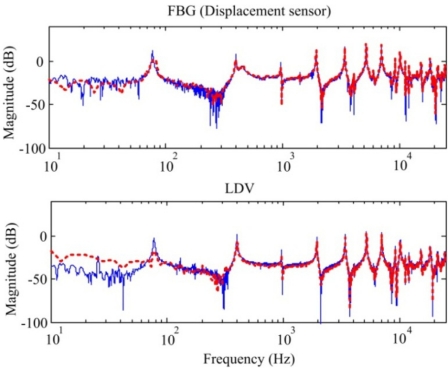
Frequency responses obtained by the FBG displacement sensor and LDV with short measurement data.

To see this effect, we excite the piezoelectric actuator again with shorter random inputs (only last for 1 s), the results obtained by the two displacement sensors are represented in Figure 18 as dashed lines. Compared with the identified frequency responses (*i.e.*, solid lines shown in Figure 18) with longer random inputs (last for 10 s), we can see that identified modes are almost the same except for the low frequencies. Repeatability of our experimental setup and the proposed FBG displacement sensor can be demonstrated in Figure 18 for frequency contents at high frequencies.

Note that the lateral mode measured in strain frequency responses shown in Figures 10 and 11 is not obtained from the displacement frequency responses (*i.e.*, Figures 17 and 18) due to the fact that the proposed FBG displacement sensor as well as the LDV is only sensitive to out-of-plane motions.

**Figure 19.**
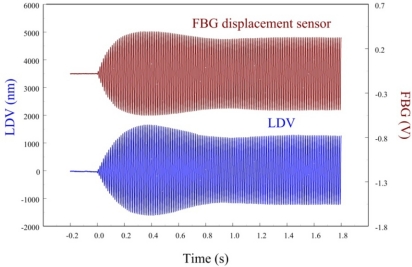
Transient displacement responses of the smart cantilever beam excited by the piezoelectric actuator at 76 Hz.

**Figure 20.**
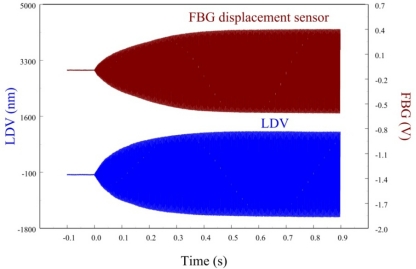
Transient displacement responses of the smart cantilever beam excited by the piezoelectric actuator at 400 Hz.

**Figure 21.**
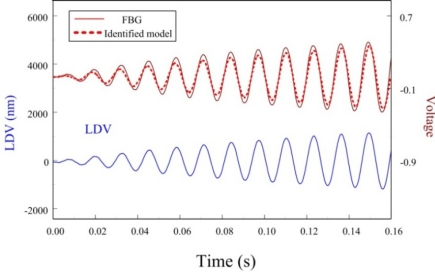
Transient displacement responses of the smart cantilever beam obtained from experiments and simulation.

In this paper, only the first two bending modes of the cantilever beam are considered and controlled by velocity feedback control. Similar to strain experiments, we also investigate dynamic sensing performances of the proposed FBG displacement sensor by measuring transient responses of the smart cantilever beam after being excited at the first two bending modes. Figures 19 and 20 are the measurement results when the smart cantilever beam is excited at 76 Hz and 400 Hz, respectively. Figure 21 demonstrates the experimental results obtained by the two displacement sensors and simulation result obtained from the identified model of the smart cantilever beam. Compared with LDV in Figures 19 to 21, we can see that the proposed FBG displacement sensor has excellent dynamic sensing performance. From Figure 21 we can also see that the proposed FBG displacement sensor is capable of being employed to perform system identification for the flexible structures.

**Figure 22.**
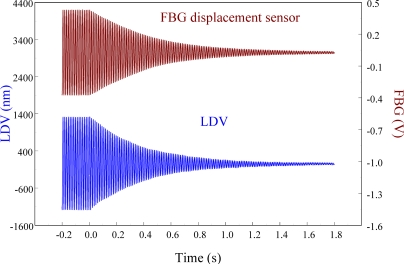
Free vibration of the smart cantilever beam excited at first resonant frequency (76 Hz) obtained from the FBG displacement sensor and LDV.

Figure 22 shows the displacement responses of the cantilever beam at first resonant frequency (*i.e.*, 76 Hz) in which excitation is removed at *t* = 0.

**Figure 23.**
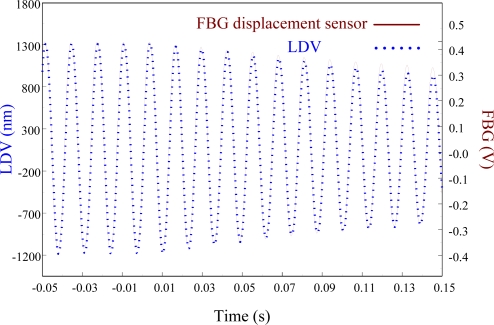
Free vibration of the smart cantilever beam excited at first resonant frequency (76 Hz) with a span of 0.2 s obtained from the FBG displacement sensor.

Figure 23 zooms in on the responses in Figure 22 and focuses on the responses within a span of 0.2 s, showing excellent agreement between two displacement sensors. The maximum peak to peak value of LDV is 2,506.44 nm. Thus, the sensitivity of the proposed FBG displacement sensor is 0.321 mV/nm by calibration with LDV.

**Figure 24.**
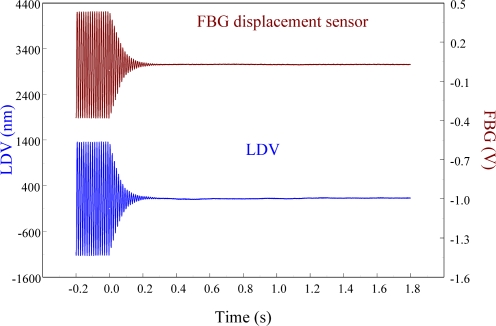
Active vibration control of the smart cantilever beam excited at first resonant frequency (76 Hz) obtained from displacement sensors.

To see the feasibility of employing the proposed FBG displacement sensor in smart structures, the velocity feedback controller is used and the control results are shown in Figure 24. It is seen that the vibrations are damped out very quickly. The settling time for the free vibration to reduce to 5% of the disturbance level is 0.15 s.

**Figure 25.**
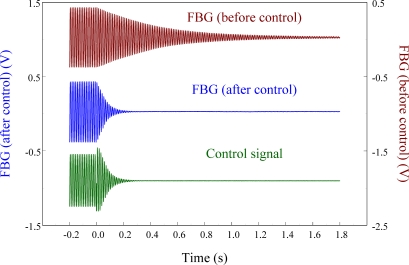
Active vibration control of the smart cantilever beam excited at first resonant frequency (76 Hz) obtained from FBG displacement sensor.

The agreement of the responses shown in Figure 24 between two sensors after the controller is applied demonstrates that the proposed out-of-plane point-wise FBG displacement is capable of being integrated into a smart structure to suppress the vibrations. According to vibration theory, damping ratio of the structure can be estimated from the log decrement:

(35) $$\varsigma =\frac{1}{2\pi n}\text{log}\left[\frac{a_{1}}{a_{n}}\right]$$

where *a_n_* is amplitude of *n* th cycle. Based on Equation (35), damping ratios of the cantilever beam excited at the first resonant frequency obtained from FBG displacement sensor without and with control are 0.0265 and 0.0042, respectively. Figure 25 shows the responses obtained from the proposed FBG displacement sensor before and after the velocity feedback controller is applied to the smart cantilever beam, with the control signal as a comparison.

**Figure 26.**
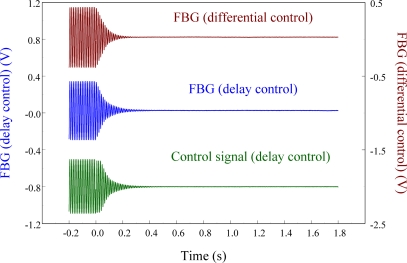
Active vibration control of the smart cantilever beam excited at first resonant frequency (76 Hz) under 3/4 phase delay control.

Next, the delay controller is applied to the system as a comparison to the velocity feedback controller. Figure 26 shows the measurement result under 3/4 phase delay control. We can see that the results obtained from differential control (*i.e.*, velocity feedback) and delay control are almost the same.

**Figure 27.**
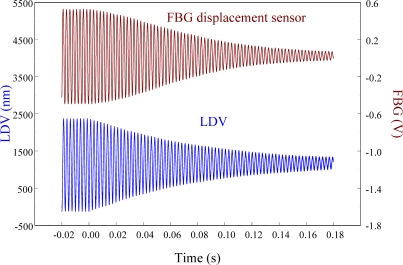
Free vibration of the smart cantilever beam excited at second resonant frequency (400 Hz).

**Figure 28.**
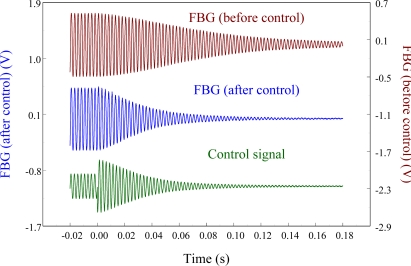
Active vibration control of the smart cantilever beam excited at second resonant frequency (400 Hz) obtained from FBG displacement sensor.

The resonant frequency of the second bending mode of the smart cantilever beam is 400 Hz. Free vibrations at 400 Hz obtained from FBG displacement sensor and LDV are shown in Figure 27 after *t* = 0. The control effect of the velocity feedback controller obtained from the FBG displacement sensor is shown in Figure 28.

**Figure 29.**
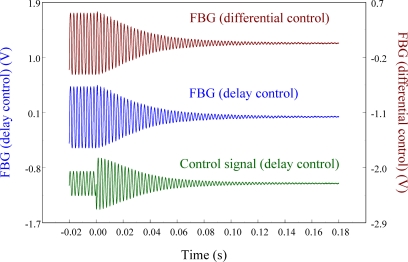
Comparison of the velocity feedback controller and delay control.

Damping ratios of the cantilever beam obtained from FBG displacement sensor before and after velocity feedback control is applied are 0.0134 and 0.0018, respectively. Similarly, the 3/4 phase delay controller is again applied to the smart cantilever beam and the comparison of two controllers (*i.e.*, differential controller and delay controller) is shown in Figure 29.

## 5. Conclusions

In this study, we investigate the feasibility of utilizing a fiber Bragg grating (FBG) displacement sensor to perform active vibration suppression of a smart cantilever beam. The set-up method proposed for the FBG displacement sensor allows the FBG to detect and feed back point-wise out-of-plane displacement responses. Before performing experiments, an optical full-field measurement technique called amplitude-fluctuation electronic speckle pattern interferometry (AF-ESPI) is used to provide full-field vibration mode shapes and resonant frequencies. Furthermore, an FBG filter-based demodulation technique is adopted to obtain high SNR and dynamic sensitivity and its demodulation performance is demonstrated by a traditional FBG strain sensor and strain gauge. Then, measurement ability of the proposed FBG displacement sensor is demonstrated by excellent agreements between experimental results obtained from the FBG displacement sensor and a laser Doppler vibrometer (LDV) sensor simultaneously. Both FBG strain and displacement sensors are utilized to perform system identification of the smart cantilever beam. Finally, a simple but effective velocity feedback control algorithm is used to verify the sensing performance of the proposed FBG displacement sensing system in an active structural control system. To our knowledge, this is the first time that a point-wise FBG displacement sensor has been integrated into a smart structure for performing active vibration control.

## Figures and Tables

**Table 1.** Parameters of the piezoceramic actuator.

Piezoceramic length	0.03 m
Piezoceramic width	0.01 m
Piezoceramic thickness *t_a_*	1 × 10^−3^ m
Piezoceramic position *x*_1_	1 × 10^−3^ m
Piezoceramic position *x*_2_	31 × 10^−3^ m
Young’s modulus *E_a_*	1,218 × 10^8^ N/m^2^
Charge constant *d*_31_	−8.6 × 10^−11^ m/V
Capacitance	4.98 nF

**Table 2.** Bending modes of the smart cantilever beam.

**Bending Mode**		
**Mode**	**Mode Shape**	**Frequency**
1	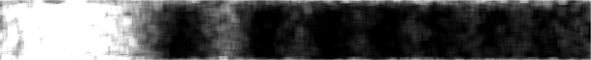	76 Hz
2	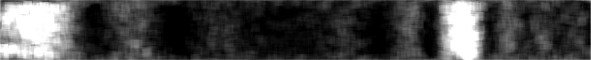	405 Hz
3	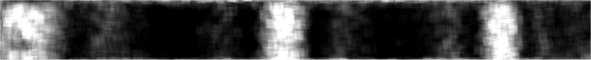	975 Hz
4	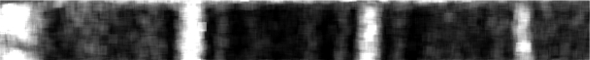	1,950 Hz
5	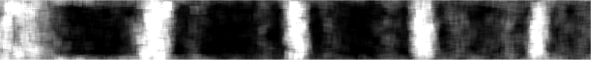	3,418 Hz
6	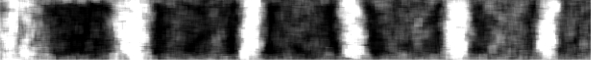	5,206 Hz
7	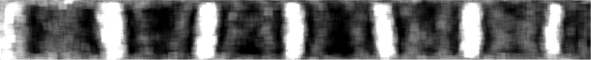	7,051 Hz
8	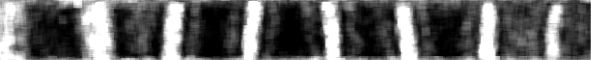	9,182 Hz
9	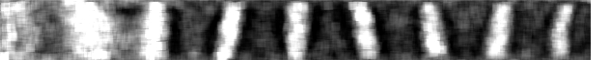	12,056 Hz
10	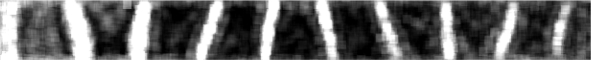	15,330 Hz

**Table 3.** Torsional modes of the smart cantilever beam.

**Torsional Mode**		
**Mode**	**Mode Shape**	**Frequency**
1	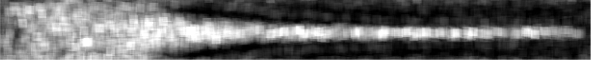	1,407 Hz
2	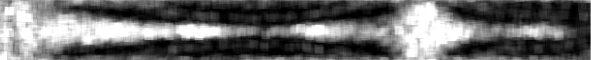	4,055 Hz
3	---	---
4	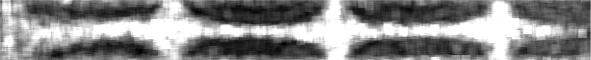	8,320 Hz
5	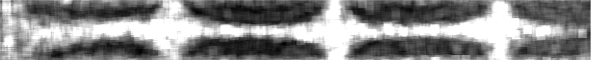	11,215 Hz
6	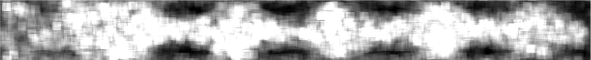	14,330 Hz
